# Editorial: Immunomodulatory Nanomaterials in Cancer Theranostics

**DOI:** 10.3389/fchem.2021.691267

**Published:** 2021-05-13

**Authors:** Sudip Mukherjee, Arindam Pramanik, Subrata Kumar Pore

**Affiliations:** ^1^ Department of Bioengineering, Rice University, Houston, TX, United States; ^2^ School of Medicine, University of Leeds, Leeds, United Kingdom; ^3^ Amity Institute of Molecular Medicine and Stem Cell Research (AIMMSCR), Amity University, Noida, India

**Keywords:** nanomaterials, immunomodulation, theranostics, drug delivery, bioimaging

The highly heterogeneous disease cancer could be successfully treated through a universal regulator i.e., immunotherapy owing to the disadvantages of traditional cancer treatments. Immunosuppression is a common phenomenon in the development of cancer and in chemotherapy especially with long-term drug treatment. Cancer immunotherapy gained huge interest among scientists last two decades. Immunotherapy can prevent, control, and eliminate all types of cancer by using the power of body’s own immune system. The main advantage is that immunotherapy could prevent the recurrence and metastasis. Different approaches of immunotherapy include cancer vaccines, targeted antibodies, oncolytic virus therapy, adaptive cell therapy and immunomodulators. Immunomodulators like cytokines, agonists, check-point inhibitors, and adjuvants can alter the immune system. There are currently hundreds of clinical trials going on with PD1/PDL1-immunotherapy alone and a few monoclonal antibody-based PD1/PDL1 checkpoint inhibitors have been approved by FDA. Despite successful implications in almost all types of cancer, there are a few limitations associated with this therapy e.g. adverse immune response like auto-immune diseases, limited infiltration of immune cells in solid tumors, immune-suppressive tumor micro-environment (TME).

Advancement of nanotechnology could overcome the limitations associated with cancer immunotherapy especially with immunomodulators. Nanomaterials (NMs) have been successfully used for targeted cancer drug delivery. In case of immunotherapy, NMs can be used both for immunosuppression for auto-immune diseases and for immunostimulant for cancers. NMs could exhibit immunostimulatory effects directly by interacting with immune cells or by delivering immunostimulatory agents like checkpoint inhibitors, adjuvants etc. in to TME. Nanomaterials are efficient for passive as well as active delivery. Passive delivery is attributed to the Enhanced Permeability and Retention effect due to the small size of NMs that can infiltrate solid tumors, remain in the circulation for long time, escape the hepatic and lymphatic clearances. On the other hand, tumor targeting active delivery can be attained by suitable surface modifications of NMs. Generally, all NMs create an immune response which can be both immunosuppressive and immunostimulatory. To reduce such adverse immune responses, NMs can be developed by suitable surface modifications, using proper preparation methods, biocompatible materials like polypeptides, proteins, DNA-fragments etc. Along with cancer immunotherapy, NMs can also be used as diagnostic tools especially for imaging.

In this special issue we hereby bring recent advancements of nanotechnology focusing on ‘immunomodulators for cancer theranostics’. The issue comprises of articles that includes reviews summarizing recent breakthrough in cancer therapeutics using immunomodulators. Alongside, it also highlights original research work to draw the attention of interested scientists working in the field of translational cancer therapy.

Jindal et al. here summarized and discussed the role of immune system to eliminate the cancer cells at the first instance but of course the cancer cells have various strategies to evade the immune system. These strategies are well described in the review by Landry et al. that described the utilization of phagocytic checkpoints, for them to be recognized as cancer therapeutics, specifically for combinational nanoparticles-based therapies. Additionally, in this special issue, authors have summarized recent developments on engineered biomaterials exhibiting immunomodulatory response thereby justifying their role in cancer theranostics. In the article by Khan et al. a detailed classification is highlighted of various nanomaterials which have been utilized as cancer immune-theranostics into organic, inorganic and their hybrids. Similar classifications and relevant analogies have been made for materials that are polymeric, metallic, lipidic or protein in nature. A detailed discussion on various physico-chemical properties of these nanoparticles such as size, surface charge, hydrophobicity was discussed by Thakur et al. in the context of immunomodulatory responses. Special highlights depicts how these nanomaterials have been utilized to target and stimulate dendritic cells, natural killer cells and T-cells to eliminate tumor cells. Aspects on biomedical applications have been explored by Khan et al. These include direct entrapment by immune cells loaded with nanomaterials -sectioned as 1) immune cell utilization 2) internalization of cytokines inside the nanoparticles for delivery of cancer cells, or delivery of antigens to cancer cells and 3) the interesting aspect of targeting the tumor microenvironment through various tumor specific receptors using their specific ligands.

Cancer imaging and the role of various nanomaterials have been thoroughly explored in this section. Khan et al. have highlighted the utilization of MRI, CT/SPECT, NIR which could be used to image cancer cells in this context. For instance, they have highlighted how gold/graphene oxide nanoparticlesconjugated with Gd^3+^ or Fe_3_O_4_-nanoparticles (NPs) have been widely used for MRI imaging of cancer cells upon targeted delivery. Likewise, tumor targeting NPs loaded with indocyanine green has been easily used for NIR imaging (Jindal et al.). Landry et al. have highlighted the outcome of various nanotheranostics clinical studies. Although it is well understood that these formulated nanotheranostics have to undergo a number of trials before they could have a direct impact on the cancer treatment but indeed there has been quite a number of promising outcomes for formulations including liposomes loaded with doxorubicin. Doxil® has been approved by FDA and EMA ([^177^Lu]-DOTA-TATE) for the treatment of neuroendocrine tumor. Likewise, gadolinium-chelated polysiloxane based nanoparticles are in phase 2 trials with quite a promising outcome. These interesting outcomes have been summarized by the authors in their review articles published under this special issue. Organometallics have also been included in this issue. Recent research work conducted by Prajapati et al. highlighted the anticancer role of copper micro sized (CuHARS) and silver nanoparticles (AgCysNPs) in glioblastoma cells. The CuHARS showed the catalytic generation of nitric oxide, which contributes to the immune system in inflammatory response. The research highlights the importance of nanohybrids as a promising tool in cancer immunotherapy.

We hope this research topic will offer researchers important information about various modern strategies of functional immunomodulatory nanoplatforms in cancer theranostics, stimulating innovative ideas for future research activities. Recent research supports the tuning of body’s immune responses to manipulate the theranostics approaches. A surge of publications and patents were observed in this hot area of cancer immunomodulation and involvement of novel materials for theranostics. However detail studies are required to establish the toxicity, degradability, pharmacokinetics and pharmacodynamics profiles of these innovative nanomaterials before their successful clinical translation. We believe we will see several clinical approved immunomodulatory drugs/nanodrugs in next few years.

